# Analysis of electrical property changes of skin by oil-in-water emulsion components

**DOI:** 10.1111/ics.12059

**Published:** 2013-06-07

**Authors:** CB Jeong, JY Han, JC Cho, KD Suh, GW Nam

**Affiliations:** * Amorepacific R&D Center, Skin Research Institute314-1, Bora-dong, Yongin-si, 446-729, Korea; † Division of Chemical Engineering, College of Engineering, Hanyang UniversitySeoul, 113-791, Korea; ‡ Department of Biomaterials Science and Engineering, College of Life Science and Biotechnology, Yonsei UniversitySeoul, 120-749, Korea

**Keywords:** corneometer, oil-in-water emulsion, poliol, relative water content increase rate, relative water contents

## Abstract

**Objectives**As the ‘Dry Skin Cycle’ produces continuous deterioration, cosmetic xerosis (flaky, dry skin) is one of the major concerns to most consumers. The purpose of this study was to investigate the moisturizing effect of oil-in-water (O/W) emulsion components. There are numerous types of oils, waxes, polyols and surfactants used as ingredients in skincare products. However, the moisturizing effect of each ingredient and understanding each use to make an effective moisturizing products are still not well understood.

**Methods** To provide answers to these questions, we investigated the moisturizing effect of widely used 41 components (four different classes) in a simple O/W emulsion using capacitance methods. 106 different single oils, and combinations of oil with oil, wax, humectants, and surfactant were formulated and tested.

**Results** In this study, we found that most of the O/W emulsion components had hydration effects on the skin. (i) The average relative water content increase (RWCI) rate of a single oil-based emulsion was 11.8 ± 5.2% (SE) and 7.9 ± 6.0% (SE) at 3 and 6 h, respectively. (ii) An oil combination emulsion showed an average RWCI rate similar to that of a single oil-based emulsion, 12.6 ± 6.0% (SE) and 12.1 ± 6.4% (SE) at 3 and 6 h, respectively (iii) A combination of waxes with oil showed an average RWCI rate of 16 ± 5.6% (SE) and 12.4 ± 4.5% (SE) at 3 and 6 h, respectively. (iv) Humectant combinations showed the highest average RWCI rate 28 ± 7.3% (SE) and 22.2 ± 7.5% (SE) at 3 and 6 h, respectively (v) Surfactant combinations had an average RWCI of 10.8 ± 4.5% (SE) and 6.0 ± 4.0% (SE) at 3 and 6 h, respectively.

**Conclusion** Interestingly, it was difficult to find moisturizing power differences among samples in the same group. Only the humectants group showed significant differences among samples. Glycerine and urea showed significant skin hydration effects compared with other humectants. We also found a significant moisturizing effect by analysing the chemical functional groups; amide class had a higher hydration effect than betaines and disaccharides in humectants combination.

Résumé

**Objectif** Puisque le «cycle de la peau sèche” produit une détérioration continue, la xérose cosmétique (squameuse, peau sèche) est l’une des préoccupations majeures pour la plupart des consommateurs. Le but de cette étude était d’étudier l’effet hydratant des composants d’émulsions H / E. Il existe de nombreux types d’huiles, des cires, de polyols, et des tensioactifs utilisés comme ingrédients dans les produits de soins de la peau. Cependant, l’effet hydratant de chaque ingrédient et de leur utilisation dans des produits hydratants efficaces ne sont pas encore bien compris.

**Methodes**Pour apporter des réponses à ces questions, nous avons étudié l’effet hydratant des 41 éléments (4 classes différentes) largement utilisés dans une émulsion simple O/W en utilisant des méthodes de capacitance. 106 huiles individuelles différentes et des combinaisons d’huile avec de l’huile, de la cire, des humectants, et de tensioactifs ont été formulées et testées.

**Resultats**Dans cette étude, nous avons constaté que la plupart des composants des émulsions huile-dans-eau (H/E) possédaient des effets d’hydratation de la peau. (i) Le taux moyen d’augmentation d’eau (RWCI = relative water content increase) d’une émulsion à base d’une seule huile était de 11,8 ± 5,2% (SE) et de 7,9 ± 6,0% (SE) à 3 et 6 h, respectivement. (ii) Une émulsion de combinaison d’huile montrait une RWCI similaire à celle d’une émulsion à base d’huile unique, 12,6 ± 6,0% (SE) et 12,1 ± 6,4% (SE) à 3 et 6 h, respectivement. (iii) Une combinaison des cires avec de l’huile présentait une RWCI de 16 ± 5,6% (SE) et 12,4 ± 4,5% (SE) à 3 et 6 h, respectivement. (iv) Les combinaisons d’humectant ont montré la plus forte augmentation avec +28 ± 7,3% (SE) et 22,2 ± 7,5% (SE) à 3 et 6 h, respectivement. (v) Les combinaisons de tensioactifs ont une RWCI moyenne de 10,8 ± 4,5% (SE) et de 6,0 ± 4,0% (SE) à 3 et 6 h, respectivement.

**Conclusion**Fait intéressant, il était difficile de trouver des différences de pouvoir d’hydratation entre les échantillons dans le même groupe. Seul le groupe des humectants a montré des différences significatives entre les échantillons. La glycérine et l’urée ont montré des effets significatifs sur l’hydratation de la peau par rapport aux autres humectants. Nous avons également constaté un effet hydratant important en analysant les groupes fonctionnels chimiques; la classe “amide” a eu un effet d’hydratation plus élevé que les bétaînes et disaccharides dans les combinaisons des humectants.

## Introduction

As the ‘Dry Skin Cycle’ produces continuous deterioration, cosmetic xerosis (interpreted as flaky, dry skin) is one of the major concerns to most consumers. When the stratum corneum (SC) barrier is dehydrated, TEWL, natural moisturizing factor (NMF) and enzymatic activity are damaged. Such damage triggers abnormal SC components, immature corneocytes, reduction in desquamatory enzyme activity and consequently induces dry skin cycle 1.

Low humidity and temperature, environmental changes, surfactant dissolution of SC lipid or NMF and ageing can affect dry skin cycle. Low humidity (<10% relative humidity) impairs the function of enzymes for the proteolysis of filaggrin and the generation of NMF. Age-related decline in NMF is caused by reduced synthesis of profilaggrin and decreased amounts of amino acids 2–3.

Dry skin cycle starts in the skin, and adequate hydration of skin is critical for maintaining its health. When skin properly maintains water, physiological factors are regulated and dry skin cycle is prevented. The ability of the skin to maintain water is primarily related to the SC, and the importance of SC water content to ‘normal’ non-flaky skin is well known 4.

Moisturizers hydrate the skin, and therefore, cosmetic companies have widely used them for dry skin treatments. A moisturizer consists of humectants, oils, lipids, aqueous materials, surfactants and other agents. Humectants attract and hold water and emollients, whereas oils and lipids occlude and hydrate the skin surface 5–6.

Previously, we proved that skin surface properties could be improved by interacting polyols and oils 7. In this study, we investigated the electrical property changes of skin by O/W emulsion components. There are numerous types of oils, waxes, polyols and surfactants used as ingredients in skincare products. However, it is not well understood how to adopt the components for making an effective moisturizing product. We investigated the moisturizing effect of 41 components (four different classes) using a non-invasive electrical capacitance method 8–9.

## Materials and methods

### Formulations of simple O/W cosmetic products

We designed a simple O/W emulsion containing a single surfactant (2.5% polyglyceryl-3 methyl glucose distearate), preservative (methylparaben, 0.2% DANISOL-M), thickener (Carbomer, 0.15% Carbopol 981) and counteragent (0.15% triethanolamine) to verify the hydration effects of each component on the human skin.

### Selecting the O/W emulsion components

To test O/W emulsion components, we divided O/W emulsion components into four categories including oils, waxes, humectants and surfactants. As we could not test all the ingredients of O/W emulsions, we chose 41 components which were widely used and could represent most of component classes. (i) Oil categories consisted of 15 different components representing six different classes. (ii) Wax categories consisted of eight different components. (iii) Humectant categories consisted of eight different components representing five different classes. (iv) Surfactant categories consisted of five different combinations of a Tween 60/Arlacel 60v system which had five different Hydrophile-Lipophile Balance (HLB) values and five different components (Table I).

**Table I:** Formulations of cosmetic products

Component group	Trade name	INCI name	Chemical class	Company	Conc. (%)
Oil	Pripure 3759	Squalane	Hydrocarbons	CRODA EUROPE LIMITED	20
	PureSyn 4	Hydrogenated Polydecene	Hydrocarbons	Sophim ExxonMobil	20
	PureSyn 150	Hydrogenated Polydecene	Hydrocarbons	ExxonMobil	20
	Panalane L 14-E	Hydrogenated Polyisobutene	Hydrocarbons	Lipo Chemicals	20
	PTO	Pentaerythrityl Tetraethylhexanoate	Esters	Nisshin oil	20
	CEH	Cetyl Ethylhexanoate	Esters	Nisshin oil	20
	Cetiol CC	Dicaprylyl carbonate	Esters	Cognis GmbH	20
	Cosmol 222	Diisostearyl Maleate	Esters	Nisshin oil	20
	O.D.O.	Caprylic/Capric Triglyceride	Triglyceride	Lasem Asia Sdn Bhd	20
	Meadowfoam Seed Oil	Limnanthes Alba (Meadowfoam) Seed Oil	Triglyceride	Natural Plant Products	20
	Amiter MA-HD	Hexyldecyl Myristoyl Methylaminopropionate	Amides	Nihon Emulsion Co., Ltd.	20
	EUTANOL G	Octyldodecanol	Alcohols	Cognis Japan Ltd.	20
	DC 345	Cyclopentasiloxane and Cyclohexasiloxane	Siloxanes and Silanes	Dow Corning	20
	DC 200 FLUID 6CS	Dimeticone	Siloxanes and Silanes	Dow Corning	20
	DC 200 FLUID 100CS	Dimeticone	Siloxanes and Silanes	Dow Corning	20
Wax	GMS 105	Glyceryl Stearate	Ester	Kwangil Co.	3
	MULTI WAX	Microcrystalline Wax	Hydrocarbons	Sonneborn	3
	Stearic acid	Stearic acid	Fatty acids	Gnam fat and oil chemical co	3
	Shea butter	Butyrospermum Parkii (Shea) Butter	Fats and oils	Sophim	3
	Cetos KD	Cetearyl Alcohol	Fatty alcohols	Gnam fat and oil chemical co	3
	Lanette 22-80	Behenyl Alcohol	Fatty alcohols	Cognis France SA	3
	Bees wax	Cera alba	Ester	Gnam fat and oil chemical co	3
	Carnauba Wax	Copernicia Cerifera (Carnauba) Wax	Ester	FONCEPI	3
Humactants	BG	Butylene Glycol	Polyols	DAICEL CEMICAL CO., LTD	8
	1,3-PG	Propylene Glycol	Polyols	DUPON	8
	NATURAL EX BP 20(AMINOCOAT)	Betaine	Betaines	DANISCO	8
	Urea	Urea	Amides	Samchun chemical CO.	8
	Konlub	PEG.PPG-17.6 Copolymer	Polymeric Ethers	KPX green chemical CO.	8
	Glycerine	Glycerine	Polyols	Household and healthcare l	8
	Trehalose	Trehalose	Disaccharides	Hayashibara	8
	DPG-FC	Dipropylene Glycol	Polyols	Dow Chemical	8
Surfactants (SFT)	Tween 60/Arlacel 60v(HLB 14.9)	polysorbate 60/sorbitan stearate		CRODA	1.5
	Tween 60/Arlacel 60v(HLB 13.0)	polysorbate 60/sorbitan stearate		CRODA	1.5
	Tween 60/Arlacel 60v(HLB 11.2)	polysorbate 60/sorbitan stearate		CRODA	1.5
	Tween 60/Arlacel 60v(HLB 9.3)	polysorbate 60/sorbitan stearate		CRODA	1.5
	Tween 60/Arlacel 60v(HLB 7.4)	polysorbate 60/sorbitan stearate		CRODA	1.5
	Montanov 202	Arachidyl alcohol and behenyl alcohol and arachidyl glucoside		SEPPIC	1.5
	Montanov 68	Cetearyl alcohol and cetearyl glucoside(20%)		SEPPIC	1.5
	Nikkomulese 41	Polyglyceryl-10 pentastearate and behenyl alcohol and sodium stearoyl lactylate		NikkoChemicals	1.5
	Tego care 450	Polyglyceryl-3 methyl glucose distearate		Evonik Industries AG	1.5
	Biophilic H	Hydrogenated lecithin and C12-16 alcohols and palmitic acid		LucasMeyer	1.5

Surfactants (2.5%): polyglyceryl-3 methyl glucose distearate; Preservative (0.2%): Methylparaben, DANISOL-M; thickener (0.15%): Carbomer, Carbopol 981; Counteragent (0.15%): Triethanolamine.

### Steps of the experiments

**Figure 1:**
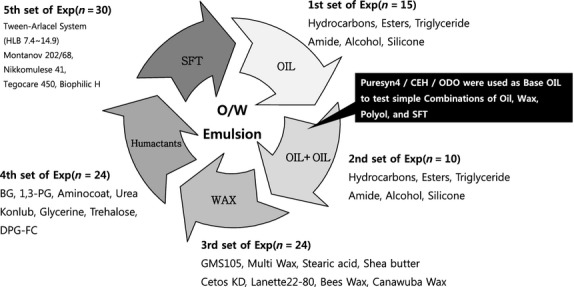
Flow of the experiments. All major components of oil-in-water (O/W) emulsion were tested from the oil to surfactants. For Testing Wax, Polyol, SFT and Polymer combination, Puresyn4 (Hydrocarbon)/CEH (Ester)/ODO (Triglyceride) were used as Base OIL.

Initial testing was conducted on 20% oil emulsions. Then, combinations of oil, wax, humectants and surfactants were formulated based on the three different oils including Puresyn 4 (hydrocarbon, hydrogenated polydecene), CEH (ester, cetyl ethylhexanoate) and O.D.O. (triglyceride, caprylic/capric triglyceride) to form O/W emulsions. Finally, we tested 106 different formulations (Fig. 1). The concentration of components in each group was determined as follows (Table I).

### Subjects and experimental design

Korean male and female volunteers (*n* = 177) in good health (20–35 years old) participated in this study. All volunteers provided written informed consent, and none of the volunteers had a history of dermatological diseases. All experiments were conducted on the skin of the ventral forearms. Volunteers were instructed not to use any cosmetic products on their ventral forearms starting 3 weeks before the experiment. To avoid environmental influences, volunteers were allowed to relax in a room maintained at a temperature of 22 ± 2°C and a relative humidity of 40 ± 2% for 30 min after washing their forearms with soap; after which, measurements of the relative water content in the SC were performed. Measurement sites for sample were changed based on circular rotation to eliminate site-to-site differences in the same anatomical region. 2 mg cm^−2^ emulsion was applied on the 3 × 3 cm site using rubber glove until emulsion was absorbed. We used four experimental sites on each forearm. Non-adsorbed materials were gently absorbed by KimWipes 5 min before the measurements to eliminate the effects of non-adsorbed materials.

### Skin capacitance measurement

The relative water content in the SC was measured with a Corneometer® CM825 (Courage + Khazaka Electronic GmbH., Cologne, Germany) based on a capacitance measurement. Corneometer was chosen to evaluate epidermal hydration changes by combinations of various emulsion components. We measured the relative water content of the experimental site 30 min after washing with soap and 3 and 6 h after application of test samples. We measured the capacitance value three times under the maximum variance 5, and the averages of the values were relative water contents. The RWCI rate was calculated by the following equation 1 10. RWCI was used to compare hydration effects among samples.

### Statistical analysis

IBM® SPSS® software version 20 (IBM-SPSS Inc. Chicago, IL, USA) and MINITAB (LEAD Technologies, Inc., Charlotte, NC, USA) Release 14.20 were used for statistical analysis. The paired *t*-test was used to compare relative water content differences at each site 0, 3 and 6 h after treatment. Differences in the hydration effect of each emulsion were analysed by ANOVA with repeated measures and ANOCOVA. A general linear model was used for component class analysis.

## Results

### Effects of by single oil (20%)-based emulsion on relative skin water content

**Figure 2:**
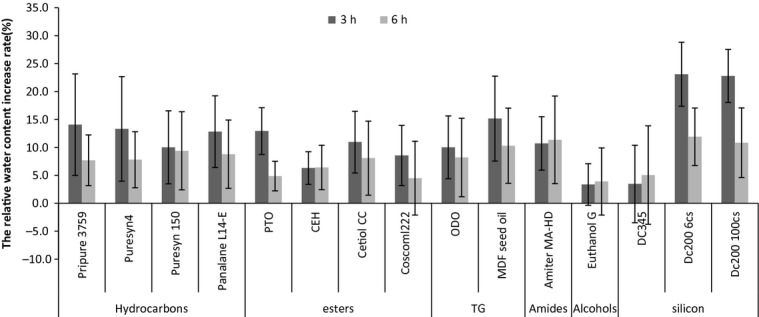
Effects of oil (20%) based emulsion on relative skin water contents increase rate.

We investigated the effects on the relative water content of skin by changing the oil part (20%) of O/W emulsions. Except for DC 345, the relative water content of skin was significantly increased during 6 h after using single oil-based emulsions. There were no significant differences among emulsions. DC200 6cs and 100cs (Dimethicone) had relatively high hydration effects on skin. DC200 6cs showed 23.1% and 11.9% RWCI rates at 3 and 6 h, respectively (Fig. 2). In most cases, the RWCI rate decreased with increasing time. However, there were no significant differences, except in the case of pentaerythrityl tetraethylhexanoate (PTO). The use of single different oil classes also had no effects on hydration.

### Effects of oil and oil combination (10 + 10%) based emulsion on relative skin water content

**Figure 3:**
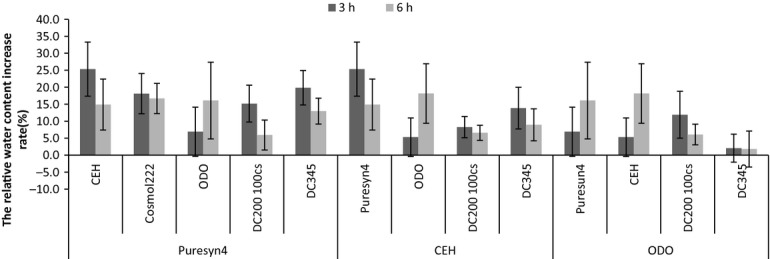
Effects of oil, oil combination (10 + 10%) based emulsion on relative skin water contents.

Combining O.D.O. (caprylic/capric triglyceride) oil with other oil classes produced no significant relative water content changes at 6 h, and Puresyn 4 (Hydrocarbon), CEH (Ester), and DC345 (cyclopentasiloxane and cyclohexasiloxane) also had no significant effect on relative water content changes at 3 h. Repeated ANOVA analysis showed there were no significant differences among oil and oil combined O/W emulsions (Table II). The RWCI rates at 3 h and 6 are shown in Fig. 3. The hydrocarbon and ester oil combination showed relatively high RWCI rates of 25.3% and 14.9%, respectively at 3 and 6 h.

**Table II:** Statistical analyses of relative water content changes by RM ANOVA

Group	Pillai’s trace	Value	*F*	Hypothesis df	Error df	Sig.*
Single oil	Wtater content	0.560	81.317	2.000	128.000	0.000***
	Water content × Single oil based emulsion group	0.226	1.174	28.000	258.000	0.256
Oil and oil combination	Water content	0.477	31.519	2.000	69.000	0.000***
	Water content× Combination of oil and oil group	0.226	0.991	18.000	140.000	0.474
Oil and wax combination	Water content	0.749	287.719	2.000	193.000	0.000***
	Water content × Combination of oil and oil group	0.182	0.847	46.000	388.000	0.751
Oil and humectants combination	Water content	0.783	352.183	2.000	195.000	0.000***
	Water content × Combination of oil and humectants group	0.538	3.139	46.000	392.000	0.000***
Oil and surfactant combination	Water content	0.771	437.757	2.000	260.000	0.000***
	Water content × Combination of oil and surfactant group	0.178	0.881	58.000	522.000	0.720

* *P* < 0.05,

*** *P* < 0.001.

### Effects of oil and wax combination (20 + 3%)–based emulsion on relative skin water content

**Figure 4:**
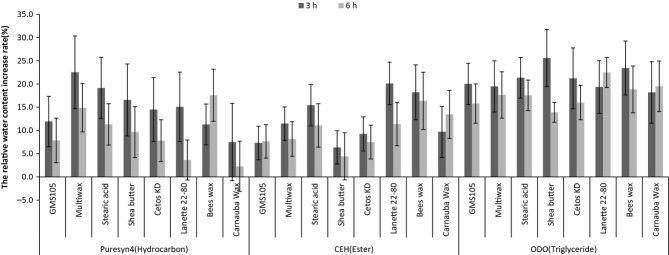
Effects of oil, wax combination (20 + 3%) based emulsion on relative skin water contents.

Most samples showed a significant difference in moisturizing effect at 3 and 6 h, but CEH (ester oil) combined with Shea butter had no significant differences at 6 h. There were no significant differences among all oils and waxes combined with O/W emulsions. The RWCI rates at 3 and 6 h are depicted in Fig. 4. ANCOVA analysis revealed that CEH (Ester) and O.D.O (Triglyceride) oil groups show significantly increased relative water contents compared with the Puresyn 4 (Hydrocarbon) oil group when combining oil with wax. However, the wax group had no differences between different classes (Table III).

**Table III:** Comparison of component class dependency on hydration by ANCOVA

Group	Component type	Time (h)	Type III sum of squares	df	Mean square	*F*	Sig.*
Oil and wax combination	Oil	3	286.849	2	143.424	4.573	0.011*
		6	128.905	2	64.453	1.774	0.172
	Wax	3	48.570	4	12.142	0.370	0.830
		6	113.887	4	28.472	0.775	0.543
Oil and humectants combination	Oil	3	133.129	2	66.565	1.029	0.359
		6	289.755	2	144.877	1.214	0.299
	Humectants	3	1705.740	4	426.435	7.334	0.000***
		6	1481.136	4	370.284	3.222	0.014*
Oil and surfactant combination	Oil	3	218.345	2	109.172	5.453	0.005**
		6	111.543	2	55.771	3.670	0.027*
	HLB	3	6.150	4	1.537	0.075	0.990
		6	29.961	4	7.490	0.426	0.790
	Surfactant	3	19.501	5	3.900	0.184	0.968
		6	40.714	5	8.143	0.593	0.706

Relative water content changes by each class were analyzed by ANCOVA. Capacitance readings at 0 time were used as a covariate.

HLB, Hydrophile-Lipophile Balance.

* *P* < 0.05,

** *P* < 0.01,

*** *P* < 0.001.

### Effects of oil and humectants combination (20 + 8%)–based emulsion on relative skin water content

**Figure 5:**
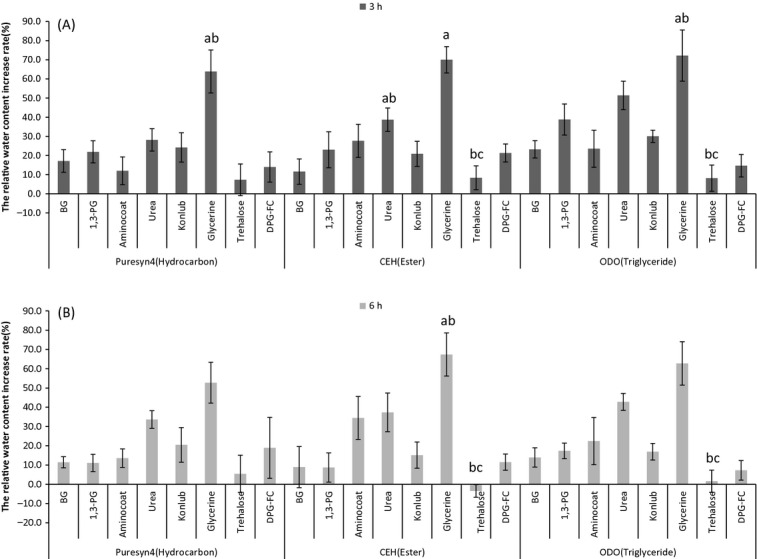
Effects of oil, humectants combination (20 + 8%) based emulsion on skin water contents. (A) Relative water content increase rate (RWCI) at 3 h (B) RWCI at 6 h; a, b, and c are significantly difference groups defined by ANCOVA.

**Figure 6:**
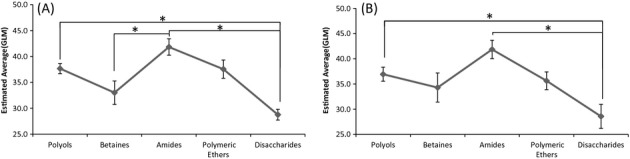
Humectants class dependent hydration effects on the skin. (A) 3 h and (B) 6 h after application of samples.

All samples based on oil and humectant combinations showed significant differences at 3 h. However, Puresyn 4 (Hydrocarbon) oil with trehalose and dipropylene glycol, CEH (Ester) oil with butylene glycol, propylene glycol, and trehalose, O.D.O. (Triglyceride) oil with betaine and trehalose showed no significant difference at 6 h. RM ANOVA showed oil and humectant combinations had significant differences among samples (Table II). Statistical analysis results of differences among samples are depicted on the RWCI rate (Fig. 5). ANCOVA analysis revealed that the hydration effect was not affected by differences of oil type, but was affected by humectant type (Table III). Statistical analysis results by ANCOVA are depicted on the RWCI rate (Fig. 5). Glycerine and urea had significant skin hydration effects compared with other humectants. The RWCI rate of glycerine was 68.7% and 61.0% and that of urea was 39.4% and 37.9%, respectively, at 3 and 6 h. When using humectant classes as an analysis category, the amide and polyol classes showed high hydration effects (Fig. 6).

### Effects of oil and surfactant combination (20 + 1.5%)–based emulsion including 3% Cetos KD (wax) as a stabilizer on relative skin water content

**Figure 7:**
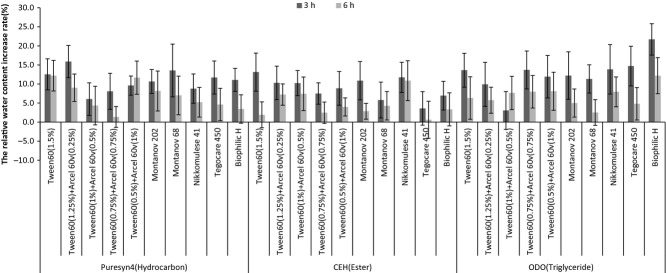
Effects of oil, surfactant combination (20 + 1.5%) based emulsion including 3% Cetos KD (wax) for stabilizing emulsion on relative skin water contents.

**Figure 8:**
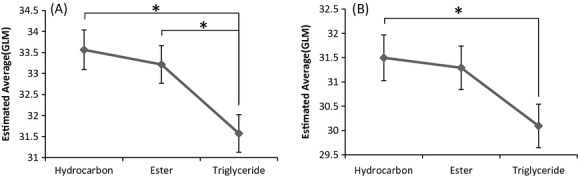
Oil class dependent hydration effects on the skin when combined with various surfactants. (A) 3 h and (B) 6 h after application of samples.

Every oil-and surfactant-based sample showed a significant moisturizing effect during 6 h. We had already determined that in all cases, an oil and wax (Cetos KD, Gnam Fat and Oil Chemical Co., Hwaseong-si, Korea) combination had a moisturizing effect during 6 h. We could therefore predict that using a different surfactant will not significantly change hydration effects. The RWCI rates at 3 and 6 h are depicted in Fig. 7. RM ANOVA/ANCOVA results also showed there were no differences among samples by using different surfactants (Tables II and III). However, the hydration effect of the combined oil and surfactant on skin was affected by using different oils. Hydrocarbon (Puresyn 4) oil showed a significant hydration effect when combined with various surfactants (Fig. 8).

## Discussion

Until now, most skin hydration research has been concerned with amelioration of dry skin or atopic dermatitis. However, repetitive usage of cleansing products such as soap also can cause dry skin symptoms by minimizing water retention by NMF, and we need to be concerned about dryness of normal skin 11. Additionally, dryness of the skin can cause continuous deterioration of the SC barrier, called the ‘Dry skin cycle’.

Maintaining an adequate hydration level in the SC is important for preventing dry skin cycle. Moisturizing cosmetics can influence skin hydration, and those cosmetic products are mainly comprised of humectants, oils, lipids and surfactants. Oils and lipids usually form the evaporation barrier film on the skin surface and humectants hold water for hydration. There are numerous cosmetic ingredients, and many of them are used as moisturizing components. However, when used as a moisturizing product containing many oils, waxes and humectants, it is hard to define each component’s hydration effects on skin. In this study, we intended to determine the skin moisturizing effect of each component based on simple differences in oils.

Our previous research focused on combinations of specific ingredients. The polyols consisted of glycerine and butylenes glycol in a ratio of 1: 1, and the oils consisted of equal parts of hydrogenated polydecene, cetyl ethylhexanoate and PTO to find the optimal hydration effect on skin 7. Even though response surface methodology analysis can reveal the optimal combination of specific components, it is hard to generalize the moisturizing effects of each O/W emulsion component.

We then designed a new experimental set for evaluating hydration effects on skin by varying oils, waxes, humectants and surfactants in an oil-in-water (O/W) emulsion based on three different oil groups. We also attempted to determine the moisturizing effect of component classes. The O/W emulsion had the composition of a conventional emulsion and consisted of 20% oil, 3% wax, 8% humectants and 1.5% surfactants. We measured hydration effect with corneometer CM825. As the capacitance methods using low frequency (40–75 Hz), the results are relatively sensitive to the dielectric constant of material. Therefore, we can conclude that values are not only directly related to SC hydration, but also that materials with varying dielectric constants could affect the results. And these results have to be considered as hydration effect measured by capacitance method 10.

Results showed that the average RWCI rate of a single oil-based emulsion was 11.8 ± 5.2% (SE) and 7.9 ± 6.0% (SE) at 3 and 6 h, respectively. An oil combination emulsion showed an average RWCI rate similar to that produced with a single oil-based emulsion, 12.6 ± 6.0% (SE) and 12.1 ± 6.4% (SE) at 3 and 6 h, respectively. Combining waxes with oil produced an average RWCI rate of 16 ± 5.6% and 12.4 ± 4.5% at 3 and 6 h, respectively. Humectant combinations showed the highest average RWCI rates of 28 ± 7.3% and 22.2 ± 7.5% at 3 and 6 h, respectively, and the surfactant combination had an average RWCI of 10.8 ± 4.5% and 6.0 ± 4.0% at 3 and 6 h, respectively.

We identified the hydration effects of each component from combinations with oils. Most oil, wax and humectant combinations with three different oils demonstrated significant moisturizing effects compared with the untreated site. However, it was difficult to identify differences in the moisturizing effect among the components. Only a small number of humectants, urea and glycerol can produce significant hydration effects compared with the effects of other components. The moisturizing effects of urea and glycerol were already well known.

Urea is significant hydrating component for the SC. Atopic dermatitis and elderly skin have deficits in urea, and dry symptoms can be corrected by topical application of urea or its precursor, arginine 12. Moreover, researchers have confirmed that topical glycerol instead of topical sebaceous lipids corrects the hydration abnormality in animal models, and endogenous glycerol has a critical role in SC hydration in humans. Researchers found that SC hydration is correlated with SC glycerol content 13–14.

We also found a significant moisturizing effect by component classes. ANCOVA analysis of the humectants class showed that the amide class had a higher hydration effect than betaines and disaccharides, and the polyol class had a higher hydration effect than disaccharides.

In this study, we attempted to determine the moisturizing effect of individual O/W emulsion components. Even though it is difficult to predict the interactions between components, we can confirm the overall hydration affect tendencies of component groups and individual ingredients when using capacitance methods (Corneometer CM825). However, those results are based on short-term changes of skin hydration, and components can have different hydration effects in long-term studies.

As a result, we can now predict the increased relative water content of skin resulting from addition of various components and recommend the moisturizer formulation for short-term moisturizing effects by capacitance method.

## Conclusion

Moisturizing effect of each O/W emulsion component and group was analysed, and results showed that most of the oils, waxes and humectants had its own moisturizing effect. There were no significant differences among components, except for the humectants group. Glycerol and urea had significant hydration effects on the skin. We can predict the increasing relative water content of skin by various components and recommend the moisturizer formulation for its short-term moisturizing effect using capacitance methods.
